# Associated factors of spontaneous reperfusion in patients with acute ST-segment elevation myocardial infarction before primary percutaneous coronary intervention

**DOI:** 10.4314/ahs.v25i2.12

**Published:** 2025-06

**Authors:** Weimin He, Yixuan Lin, Fengxin Yuan, Tong Zhang

**Affiliations:** Department of Cardiology, The Sixth Affiliated Hospital, South China University of Technology, Foshan, China

**Keywords:** Acute ST-segment elevation myocardial infarction, spontaneous reperfusion, preoperative factors

## Abstract

**Background:**

This is a retrospective analysis of the relationship between preoperative factors and spontaneous reperfusion (SR) in infarct-related arteries (IRAs) in patients with ST-segment elevation myocardial infarction (STEMI) undergoing percutaneous coronary intervention (PCI).

**Methodology:**

834 patients who were classified into the SR group (TIMI flow grade 2-3) and non-spontaneous reperfusion (NSR) group (TIMI flow grade 0-1) based on coronary angiography. The baseline characteristics and preoperative factors of the two groups were analyzed.

**Results:**

Compared with the NSR group, the SR group had shorter time from symptom onset to first medical contact (P=0.002) and from symptom onset to antiplatelet loading (P=0.003), however, there was no statistically significant difference in the mode of admission and loading of antiplatelet drugs between the two groups. Multivariate logistic regression analysis indicated that diabetes history, smoking history, triglyceride level, time from symptom onset to first medical contact, time from symptom onset to antiplatelet loading, and time from antiplatelet loading to coronary angiography were all factors influencing SR were all factors influencing SR.

**Conclusion:**

The occurrence of SR is related to early medical contact and timely use of antiplatelet drugs. Patients with diabetes history, smoking history, higher triglyceride level may benefit more.

## Introduction

ST-segment elevation myocardial infarction (STEMI) refers to myocardial ischemia and necrosis caused by thrombotic obstruction, resulting from the rupture of atherosclerotic plaques in the coronary artery^1^. It is a major cause of death and disability in China. Early opening of the infarct-related artery (IRA) and achieving myocardial reperfusion can salvage surviving myocardium, reduce mortality, and improve the short-term and long-term prognosis of patients^2^,^3^. Direct percutaneous coronary intervention (PCI) is the preferred myocardial reperfusion therapy for STEMI patients within 12 hours of onset^4^. During emergent coronary angiography, some STEMI patients had varying degrees of restored blood flow in the IRA, a phenomenon referred to as spontaneous reperfusion (SR). SR of the IRA is equivalent to achieving myocardial reperfusion on its own. STEMI patients with SR have a smaller infarct size, lower peak levels of myocardial biomarkers, higher left ventricular ejection fraction, lower incidence of congestive heart failure, and lower incidence of recurrent acute coronary syndrome, so SR is an independent predictor of short-term and long-term survival in STEMI patients^5^. The mechanisms behind SR of the IRA are still unclear and may be related to endogenous thrombolysis^6^. Some clinical factors associated with SR have been reported, including gender, age, hypertension, diabetes, smoking history, blood lipid levels, and homocysteine, but the conclusions are not consistent^7^-^10^, and those factors are intervenable. The aim of this study was to investigate intervenable factors that could increase the occurrence of SR between the onset of myocardial infarction symptoms and coronary angiography. The findings of this study ultimately provide valuable references for clinical treatment and prognosis improvement in STEMI patients.

## Patients

This retrospective analysis was conducted on patients with acute STEMI who underwent primary PCI at the Sixth Affiliated Hospital, South China University of Technology from January 1, 2016 to March 31, 2020. Inclusion criteria were as follows: patients (≥18 years old) with acute STEMI who were diagnosed within 12 hours and underwent primary PCI according to the guidelines for the diagnosis and treatment of acute STEMI in China (2019)^4^. Exclusion criteria were as follows: patients whose onset of symptoms was more than 12 hours ago, those who had undergone thrombolytic therapy, those who had developed cardiogenic shock or heart failure and required the use of an intra-aortic balloon pump or mechanical ventilation before the operation, those who were diagnosed with non-coronary artery obstruction myocardial infarction or diagnosed with non-coronary artery disease (such as myocarditis, aortic dissection, etc.) after the operation. All patients received a loading dose of dual antiplatelet therapy (aspirin 300 mg + clopidogrel 600 mg or ticagrelor 180 mg) before the primary PCI, and 2,000 U of heparin was intravenously injected after successful puncture for coronary angiography. All the patients won't received any other drugs or interventions before PCI. This was a retrospective study with anonymous data. The study applied to the Medical Ethics Committee of Nanhai District People's Hospital for exemption of patient informed consent and was approved.

## Research Methods

According to the Thrombolysis in Myocardial Infarction (TIMI) flow grade during emergency coronary angiography, the TIMI 2 and 3 flow grades were classified as the spontaneous reperfusion group (SR group), while the TIMI 0 and 1 flow grades were classified as the non-spontaneous reperfusion group (NSR group). The TIMI flow grade criteria are as follows: grade 0 indicates no blood flow beyond the occlusion, grade 1 indicates incomplete filling of the distal vessel beyond the occlusion, grade 2 indicates partial filling of the distal vessel beyond the occlusion within 3 cardiac cycles, and grade 3 indicates complete filling of the distal vessel beyond the occlusion within 3 cardiac cycles^11^. The assessment of TIMI blood flow was conducted by two primary surgeons in our department during the procedure, and we would check the evaluation records again in the retrospective analysis.

The baseline characteristics of the two groups were compared, including age, gender, history of hypertension, history of diabetes, smoking history, lipid levels (including low-density lipoprotein cholesterol, high-density lipoprotein cholesterol, triglycerides, and lipoprotein (a)), serum creatinine, and culprit vessel (inferred based on ST-segment elevation on electrocardiography). The interventions before PCI in the two groups of patients were analyzed, including (1) mode of presentation (e.g., direct admission to our hospital, transferred from another hospital, etc.), (2) antiplatelet loading drugs (clopidogrel or ticagrelor), and (3) timing of interventions, such as the time from symptom onset to the first medical contact, the time from the first medical contact to dual antiplatelet loading, the time from symptom onset to dual antiplatelet loading.

## Statistical analysis

Data were analyzed using Statistical Product and Service Solutions (SPSS) 23.0 (IBM, Armonk, NY, USA). Normally distributed continuous variables were expressed as means ± standard deviations, and between-group comparisons were performed using independent-sample t-tests. Non-normally distributed variables were expressed as medians and interquartile ranges, and between-group comparisons were performed using non-parametric tests. Count data were expressed as ratios or constituent ratios, and between-group comparisons were performed using chi-square tests. A multivariate logistic regression model was used to analyze the impact of various factors on SR. A P-value less than 0.05 was considered statistically significant.

## Results

### Baseline characteristics

A total of 834 patients were included in this study, of which 197 were in the SR group and 637 were in the NSR group. There were no statistically significant differences between the two groups in baseline characteristics, including age, gender, history of hypertension, history of smoking, low-density lipoprotein, high-density lipoprotein, lipoprotein(a), creatinine, and culprit vessel. The proportion of patients with diabetes (20.30% vs. 13.34%, P=0.017) and the level of triglyceride (2.29±2.21 mmol/L vs. 1.87±1.47 mmol/L, P=0.004) were higher in the SR group than in the NSR group (Table 1).

**Table 1:** Baseline characteristics between the two groups

Items	SR group n=197	NSR group n=637	P
Age, years	57.39±14.20	57.08±14.07	0.791
Sex			0.207
Man, n (%)	173(87.82)	536(84.14)	
Female, n (%)	24(12.18)	101(15.86)	
Hypertension, n (%)	87(44.16)	253(39.72)	0.267
Diabetes, n (%)	40(20.30)	85(13.34)	0.017
Smoke, n (%)	111(56.34)	328(51.49)	0.233
LDL_C(mmol/L)	2.74±1.21	2.70±1.18	0.663
HDL_C(mmol/L)	1.20±0.28	1.23±0.30	0.230
TG(mmol/L)	2.29±2.21	1.87±1.47	0.004
Lipoprotein a(mg/dl)	40.86±33.01	42.43±32.87	0.578
Cr(umol/L) culprit artery	88.20±26.77	75.83±19.56	0.212
LAD, n (%)	110(55.84)	321(50.39)	0.181
RCA, n (%)	65(32.99)	244(38.30)	0.177
LCX, n (%)	22(11.17)	72(11.30)	0.958

LAD: left anterior descending coronary artery; RCA: right coronary artery; LCX: left circumflex coronary artery.

### Preoperative factors in SR and NSR groups

Compared with the NSR group, the SR group had a shorter time from symptom onset to first medical contact (108.86±101.47 mins vs. 139.21±128.30 mins, P=0.002), a shorter time from symptom onset to dual antiplatelet therapy (129.13±108.59 mins vs. 161.78±129.69 mins, P=0.003). There were no statistically significant differences in the other time points. There were no statistically significant differences between the two groups in terms of hospital admission (directly admission vs. transferred) and antiplatelet drug therapy (clopidogrel vs. ticagrelor). (Table 2).

**Table 2:** Preoperative data between the two groups

Preoperative condition	SR group n=197	NSR group n=637	P
mode of admission			0.258
Direct to our hospital, n (%)	42(21.32)	161(25.27)	
Transferred from other hospital, n (%)	155(78.68)	476(74.73)	
Loading antiplatelet drugs			0.491
Clopidogrel, n (%)	61(30.96)	181(28.41)	
Ticagrelor, n (%)	136(69.04)	456(71.59)	
time points			
Symptom onset - First medical contact (mins)	108.86±101.47	139.21±128.30	0.002
First Medical Contact - loading antiplatelet (mins)	20.27±18.88	22.57±21.57	0.215
Symptom onset - loading antiplatelet (mins)	129.13±108.59	161.78±129.69	0.003
Loading antiplatelet - Coronary angiography (mins)	108.94±77.63	91.91±53.42	0.021
Symptom onset - Coronary angiography (mins)	238.07±131.73	253.69±142.70	0.204

### Comparison of time points by mode of admission

To investigate the differences in time points between patients with different hospital admission methods, patients were divided into two groups: directly to our hospital and transferred from another hospital. The time from symptom onset to first medical contact (162.39±139.94 mins vs. 122.15±116.05 mins, P<0.001) and the time from symptom onset to dual antiplatelet therapy (178.94±141.76 mins vs. 146.54±118.21 mins, P=0.003) were both longer in the directly to our hospital group than in the transferred from another hospital group. However, the time from dual antiplatelet therapy to coronary angiography was significantly shorter in the directly to our hospital group than in the transferred from another hospital group (62.56±26.80 mins vs. 108.71±64.12 mins, P<0.001). (Table 3).

**Table 3:** Time points grouped by mode of admission

Time points	Direct to our hospital n=203	Transferred from other hospitals n=631	P
Symptom onset - First medical contact (mins)	162.39±139.94	122.15±116.05	<0.001
First Medical Contact - Loading antiplatelet (mins)	16.55±12.60	24.39±20.99	<0.001
Symptom onset - Loading antiplatelet (mins)	178.94±141.76	146.54±118.21	0.003
Loading antiplatelet - Coronary Angiography (mins)	62.56±26.80	108.71±64.12	< 0.001
Symptom onset - Coronary Angiography (mins)	241.51±142.52	255.24±136.88	0.27

### Analysis of Subgroups of Patients Transferred from Other Hospitals

Due to the significantly longer time of “dual antiplatelet loading-coronary angiography” for patients transferred from other hospitals compared to those directly admitted to our hospital, subgroup analysis was conducted for these patients in this study. A total of 631 patients were included in the subgroup analysis, among whom 154 were in the SR group and 477 were in the NSR group. There were no significant differences in age, gender, history of hypertension, low-density lipoprotein, high-density lipoprotein, lipoprotein(a), or culprit vessels between the two groups. The proportion of patients with a history of diabetes (19.84% vs. 13.00%, P=0.047), smoking history (61.69% vs. 50.94%, P=0.020), and triglycerides (2.37±2.32 mmol/L vs. 1.92±1.53mmol/L, P=0.011) were significantly higher in the SR group than in the NSR group. In terms of time points, the “symptom onset-first medical contact” (94.58±88.87mins vs. 130.70±122.10mins, P=0.002) and “symptom onset-antiplatelet loading” (116.32±94.86 mins vs. 155.91±123.18 mins, P=0.001) were significantly shorter in the SR group than in the NSR group, while there was no significant difference in the “antiplatelet loading-coronary angiography” time between the two groups (SR 117.03±84.74 mins vs. NSR 106.12±56.10 mins, P=0.097). (Table 4).

**Table 4:** Data analysis of subgroups transferred from other hospitals

Items	SR group n=154	NSR group n=477	P
Age, years	56.15±14.38	56.50±13.53	0.785
Sex			0.106
Man, n (%)	139(90.26)	406(85.12)	
Female, n (%)	15(9.74)	71(14.88)	
Hypertension, n (%)	66(42.86)	186(38.99)	0.395
Diabetes, n (%)	30(19.48)	62(13.00)	0.047
Smoke, n (%)	95(61.69)	243(50.94)	0.020
LDL_C(mmol/L)	2.68±1.15	2.68±1.17	0.993
HDL_C(mmol/L)	1.18±0.28	1.22±0.30	0.140
TG(mmol/L)	2.37±2.32	1.92±1.53	0.011
Lipoprotein a(mg/dl) culprit artery	41.47±33.48	42.54±33.14	0.739
LAD, n (%)	85(55.19)	240(50.31)	0.292
RCA, n (%)	49(31.82)	183(38.36)	0.143
LCX, n (%) time points	22(14.29)	72(15.09)	0.806
Symptom onset - First medical contact (mins)	94.58±88.87	130.70±122.10	0.002
First Medical Contact - Loading antiplatelet (mins)	21.74±19.15	25.21±21.49	0.107
Symptom onset -Loading antiplatelet (mins)	116.32±94.86	155.91±123.18	0.001
Loading antiplatelet - Coronary Angiography (mins)	117.03±84.74	106.12±56.10	0.097
Symptom onset - Coronary Angiography (mins)	233.35±129.53	262.03±138.53	0.023

**Figure 1:**
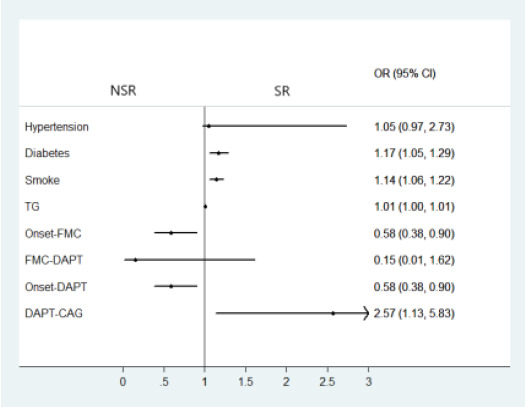
Multivariate logistic regression analysis of some factors of the subgroup transferred from other hospitals

A multivariate logistic regression model was used to analyze the effects of a history of hypertension, diabetes, smoking, triglyceride levels, time from symptom onset to first medical contact, time from first medical contact to double antiplatelet loading, time from symptom onset to double antiplatelet loading, and time from double antiplatelet loading to coronary angiography on SR in the subgroup of patients transferred from other hospitals. The results showed that diabetes history (OR 1.166, 95%CI 1.053-1.290), smoking history(OR 1.139, 95%CI 1.060-1.224), triglyceride level(OR 1.007, 95%CI 1.000-1.013), time from symptom onset to first medical contact(OR 0.584, 95%CI 0.379-0.900), time from symptom onset to antiplatelet loading(OR 0.584, 95%CI 0.379-0.900), and time from antiplatelet loading to coronary angiography (OR 2.568, 95%CI 1.132-5.829) were all factors influencing SR. (Table 5, Figure 1).

**Table 5:** Multivariate logistic regression analysis of some factors of the subgroup transferred from other hospitals

Factor	OR	95% CI [lower, upper]	P
Hypertension	1.045	[0.970, 2.727]	0.242
Diabetes	1.166	[1.053, 1.290]	0.003
Smoke	1.139	[1.060, 1.224]	<0.001
TG	1.007	[1.000, 1.013]	0.004
Symptom onset - First medical contact	0.584	[0.379, 0.900]	0.015
First Medical Contact – Loading antiplatelet	0.152	[0.014, 1.615]	0.118
Symptom onset - Loading antiplatelet	0.584	[0.379, 0.900]	0.015
Loading antiplatelet - Coronary Angiography	2.568	[1.132, 5.829]	0.024

## Discussion

Different studies have different diagnostic criteria for SR, with most studies using the TIMI flow grade based on the culprit artery in coronary angiography. Previous evidence defined SR as TIMI 312, Christian et al.^13^ defined it as TIMI 1-3, and Lee et al.^14^ defined it as TIMI 2-3. The reported incidence of SR in most studies is between 13.3% and 22.4%^12^-^16^. In this study, SR was defined as TIMI 2-3 during angiography, and the incidence was 23.62%, which is similar to previous studies. Previous research has shown that STEMI patients with SR have a smaller infarct size, lower peak levels of myocardial biomarkers, higher left ventricular ejection fraction, lower incidence of congestive heart failure, and lower incidence of recurrent acute coronary syndrome^17^. These studies demonstrate that early reopening of the IRA and achieving myocardial reperfusion can salvage viable myocardium, reduce infarct size, lower mortality rates, and improve both short-term and long-term outcomes for patients 18. The CADIL-LAC and HORIZONS-AMI trials also demonstrated that, even after adjusting for the impact of PCI procedures, patients with SR had a 39% lower mortality rate at 1 year compared to those without SR^19^. In recent years, there have been many studies on predictive factors for SR, including gender, age, hypertension, diabetes, smoking history, lipid levels, homocysteine levels, monocyte levels, and fibrinogen^7^-^10^. However, some of these factors cannot be intervened (such as gender and age), and some cannot be temporarily intervened before surgery (such as hypertension, diabetes, smoking, and lipids). To date, there is no research on factors that can be temporarily intervened to increase the likelihood of SR occurring between the onset of myocardial infarction symptoms and coronary angiography.

In this study, grouping analysis based on the occurrence of STEMI was conducted, and it was found that the mode of medical treatment (direct admission to our hospital or transfer from an external hospital) and antiplatelet drugs (clopidogrel or ticagrelor) were not related to the occurrence of STEMI. The time from symptom onset to the first medical contact, the time from symptom onset to the administration of dual antiplatelet loading, and the time from administration of dual antiplatelet loading to coronary angiography were all shorter in the SR group than in the NSR group, suggesting that the occurrence of STEMI may be related to early medical contact, early administration of anti-platelet drugs, and the duration of action of antiplatelet drugs. Based on the mode of medical treatment, it was found that patients who directly admitted to our hospital could be diagnosed and treated with antiplatelet drugs more quickly than those who transferred from an external hospital, but the time from onset of symptoms to the first medical contact was longer for patients who directly admitted to our hospital (possibly due to some patients choosing to travel to a higher-level hospital instead of seeking medical attention nearby), and the duration of action of antiplatelet drugs was shorter, resulting in no difference in the incidence of STEMI between the two groups. To eliminate the interference of antiplatelet drugs duration of action, subgroup analysis was performed on patients transferred from external hospitals. The patients in this subgroup were divided into SR and NSR groups, and there were no statistically significant differences in general information such as age, sex, history of hypertension, low-density lipoprotein, high-density lipoprotein, and culprit vessel between the two groups. The time interval from administration of dual antiplatelet therapy to coronary angiography was also similar between the two groups, indicating comparability. In this subgroup analysis, the time from symptom onset to the first medical contact and the time from symptom onset to the administration of dual antiplatelet therapy were both longer in the SR group than in the NSR group, consistent with the results of the entire population study, once again indicating that early medical contact and administration of antiplatelet drugs can increase the incidence of SR. Therefore, for patients with symptoms of myocardial infarction, early diagnosis and treatment in nearby hospitals and administration of antiplatelet drugs may be a feasible way to improve the outcome of STEMI patients, and the use of wearable devices for remote transmission and diagnosis of electrocardiograms may be further explored to verify this possibility.

The subgroup analysis of patients transferred from the other hospitals had a higher incidence of diabetes, smoking history, and elevated triglyceride levels in SR group compared to the NSR group. Previous studies have shown that high blood glucose levels have a negative impact on platelet function and the fibrinolytic system^20^, long-term smoking increases the level of platelet-derived growth factor, leading to platelet activation and production^21^, and it can serve as an independent predictor of IRA patency^18^,^22^. Hypertriglyceridemia can lead to excessive platelet activation^23^. Previous studies have shown that the composition of coronary artery thrombi in STEMI patients differs at different time intervals of ischemia. In patients with ischemia time ≤ 4 hours, the thrombi are mainly composed of white thrombi, which are predominantly platelet-based, while in patients with ischemia time of 4-7 hours, the thrombi are mainly composed of mixed thrombi. White thrombi are mainly composed of platelets, so early antiplatelet therapy has a significant effect on thrombolysis and SR^24^. Therefore, although diabetes, smoking, and high triglycerides may not individually serve as predictors of SR in STEMI patients, patients with these factors may benefit more from early antiplatelet therapy, thereby increasing the incidence of SR.

## Conclusion

SR is associated with early medical contact, early use of antiplatelet drugs, and the time of action after taking antiplatelet drugs. Early use of antiplatelet drugs can increase the incidence of SR. Patients should seek medical attention as soon as possible and receive antiplatelet therapy after experiencing myocardial infarction symptoms. Remote wearable electrocardiogram devices may further advance the timing of antiplatelet drug administration. STEMI patients with a history of diabetes, smoking, and hypertriglyceridemia may derive greater benefits from early antiplatelet therapy.

## Limitations

This study is a single-center retrospective analysis, and there may be systematic biases in the case data, which require multi-center data to further confirm the study's conclusions, and it may reveal the potential therapeutic prospects of remote wearable electrocardiogram monitoring devices. In this study, there may be some differences between the evaluation of TIMI blood flow by the surgical staff and the evaluation in the laboratory, and further correction of TIMI frame count and thrombus score data is needed. This study did not clarify the causal relationship between diabetes, smoking, triglycerides, and SR, and further research is needed to clarify their relationship which may indicate the risk factors and mechanisms of thrombosis more clear.

## References

[1] Hu S, Yang Y, Liu L (2019). [Summary of the 2018 Report on Cardiovascular Diseases in China]. Chinese Circukiticn Journal.

[2] Puymirat E, Simon T, Steg PG, Schiele F, Gueret P, Blanchard D (2012). Association of changes in clinical characteristics and management with improvement in survival among patients with ST-elevation myocardial infarction. JAMA-J Am Med Assoc.

[3] Gale CP, Allan V, Cattle BA, Hall AS, West RM, Timmis A (2014). Trends in hospital treatments, including revascularisation, following acute myocardial infarction, 2003-2010: a multilevel and relative survival analysis for the National Institute for Cardiovascular Outcomes Research (NICOR). Heart.

[4] (2019). [2019 Chinese Society of Cardiology (CSC) guidelines for the diagnosis and management of patients with ST-segment elevation myocardial infarction]. Zhonghua Xin Xue Guan Bing Za Zhi.

[5] Rimar D, Crystal E, Battler A, Gottlieb S, Freimark D, Hod H (2002). Improved prognosis of patients presenting with clinical markers of spontaneous reperfusion during acute myocardial infarction. Heart.

[6] Zhao R, Yu J, Yan L (2012). Clinical analysis on acute ST-segment elevation myocardial infarction in patients with normal coronary angiography. Chin J Intervent Cardiol.

[7] Kim JW, Seo HS, Suh SY, Choi CU, Kim EJ, Rha SW (2008). Relationship between lipoprotein(a) and spontaneous recanalization of infarct-related arteries in the early phase of acute myocardial infarction. Clin Cardiol.

[8] Li J, Zhou Y, Zhang Y, Zheng J (2018). Admission homocysteine is an independent predictor of spontaneous reperfusion and early infarct-related artery patency before primary percutaneous coronary intervention in ST-segment elevation myocardial infarction. BMCmc Cardiovasc Disor.

[9] Zhao YP, Ji YY, Wang FY, Wang SL, Lai GK, Wang T (2019). [Value of fibrinogen to albumin ratio on predicting spontaneous recanalization of infarct-related artery in patients with acute ST-segment elevation myocardial infarction]. Zhonghua Xin Xue Guan Bing Za Zhi.

[10] Wang J, He SY (2020). Clinical and angiographic characteristics of patients with spontaneous reperfusion in ST-segment elevation myocardial infarction. Medicine.

[11] Bainey KR, Fu Y, Wagner GS, Goodman SG, Ross A, Granger CB (2008). Spontaneous reperfusion in ST-elevation myocardial infarction: comparison of angiographic and electrocardiographic assessments. Am Heart J.

[12] Steg PG, Himbert D, Benamer H, Karrillon G, Boccara A, Aubry P (1997). Conservative management of patients with acute myocardial infarction and spontaneous acute patency of the infarct-related artery. Am Heart J.

[13] Christian TF, Milavetz JJ, Miller TD, Clements IP, Holmes DR, Gibbons RJ (1998). Prevalence of spontaneous reperfusion and associated myocardial salvage in patients with acute myocardial infarction. Am Heart J.

[14] Lee CW, Hong MK, Lee JH, Yang HS, Kim JJ, Park SW (2001). Determinants and prognostic significance of spontaneous coronary recanalization in acute myocardial infarction. Am J Cardiol.

[15] Ross AM, Coyne KS, Reiner JS, Greenhouse SW, Fink C, Frey A (1999). A randomized trial comparing primary angioplasty with a strategy of short-acting thrombolysis and immediate planned rescue angioplasty in acute myocardial infarction: the PACT trial. PACT investigators. Plasminogen-activator Angioplasty Compatibility Trial. J Am Coll Cardiol.

[16] Stone GW, Cox D, Garcia E, Brodie BR, Morice MC, Griffin J (2001). Normal flow (TIMI-3) before mechanical reperfusion therapy is an independent determinant of survival in acute myocardial infarction: analysis from the primary angioplasty in myocardial infarction trials. Circulation.

[17] Fefer P, Hod H, Hammerman H, Boyko V, Behar S, Matetzky S (2009). Relation of clinically defined spontaneous reperfusion to outcome in ST-elevation myocardial infarction. Am J Cardiol.

[18] Rakowski T, Dudek D, Dziewierz A, Yu J, Witzenbichler B, Guagliumi G (2013). Impact of infarct-related artery patency before primary PCI on outcome in patients with ST-segment elevation myocardial infarction: the HORIZONS-AMI trial. Euro Intervention.

[19] Brener SJ, Mehran R, Brodie BR, Guagliumi G, Witzenbichler B, Cristea E (2011). Predictors and implications of coronary infarct artery patency at initial angiography in patients with acute myocardial infarction (from the CADILLAC and HORIZONS-AMI Trials). Am J Cardiol.

[20] Gresele P, Guglielmini G, De Angelis M, Ciferri S, Ciofetta M, Falcinelli E (2003). Acute, short-term hyperglycemia enhances shear stress-induced platelet activation in patients with type II diabetes mellitus. J Am Coll Cardiol.

[21] Lupia E, Bosco O, Goffi A, Poletto C, Locatelli S, Spatola T (2010). Thrombopoietin contributes to enhanced platelet activation in cigarette smokers. Atherosclerosis.

[22] Rakowski T, Dziewierz A, Siudak Z, Mielecki W, Bierca K, Legutko J (2011). Predictors of infarct-related artery patency following combined lytic therapy in patients with ST-segment elevation myocardial infarction treated with immediate percutaneous coronary intervention. Kardiol Pol.

[23] Wang T, Xu J, Fu L, Li L (2020). Hypertriglyceridemia is associated with platelet hyperactivation in metabolic syndrome patients. Int J Clin Pract.

[24] Rao M, Zhao B, Liu P (2017). Pathological analysis of coronary artery thrombus in different ischemic time in patients with ST-segment elevation acute myocardial infarction. Med J Chin PLA.

